# A Raster-Based Subdividing Indicator to Map Urban Heat Vulnerability: A Case Study in Sydney, Australia

**DOI:** 10.3390/ijerph15112516

**Published:** 2018-11-09

**Authors:** Wei Zhang, Phil McManus, Elizabeth Duncan

**Affiliations:** 1 School of Geographical Sciences, Southwest University, Chongqing 400715, China; 2 Research Center of Urban and Regional Planning in Southwest China, Chongqing 400715, China; 3 School of Geosciences, The University of Sydney, Camperdown, NSW 2006, Australia; phil.mcmanus@sydney.edu.au (P.M.); elizabeth.duncan@sydney.edu.au (E.D.)

**Keywords:** heat vulnerability, indicators, mapping, demography, public health, Sydney

## Abstract

Assessing and mapping urban heat vulnerability has developed significantly over the past decade. Many studies have mapped urban heat vulnerability with a census unit-based general indicator (CGI). However, this kind of indicator has many problems, such as inaccurate assessment results and lacking comparability among different studies. This paper seeks to address this research gap and proposes a raster-based subdividing indicator to map urban heat vulnerability. We created a raster-based subdividing indicator (RSI) to map urban heat vulnerability from 3 aspects: exposure, sensitivity and adaptive capacity. We applied and compared it with a raster-based general indicator (RGI) and a census unit-based general indicator (CGI) in Sydney, Australia. Spatial statistics and analysis were used to investigate the performance among those three indicators. The results indicate that: (1) compared with the RSI framework, 67.54% of very high heat vulnerability pixels were ignored in the RGI framework; and up to 83.63% of very high heat vulnerability pixels were ignored in the CGI framework; (2) Compared with the previous CGI framework, a RSI framework has many advantages. These include more accurate results, more flexible model structure, and higher comparability among different studies. This study recommends using a RSI framework to map urban heat vulnerability in the future.

## 1. Introduction

Heat waves are becoming a significant public health concern. Long exposure to extreme heat can severely affect a person’s physiological comfort, resulting in heat stress and, in extreme cases, death [1,2,3]. In 2006, the California heat wave resulted in 16,000 additional emergency room visits and 1200 additional hospitalizations [4]; In 2003, unprecedented heat waves in Western Europe resulted in 70,000 excess deaths; and the 2010 heat wave in Russia caused an estimated 55,000 deaths [5]. In Australia, heat waves have caused more deaths than any other natural hazard, except disease, over the past 200 years [6]. More than 4000 deaths have been attributed to heat waves since 1800 [7], twice the number of deaths due to either flood or cyclones [6]. 

Heat waves are of particular concern in large urban areas, where there are population concentrations and where the urban heat island (UHI) effect exacerbates temperatures [8,9]. The urban heat is superimposed on the background mesoscale climate, which exacerbates heat exposure and increases the risk of mortality and morbidity in cities [10,11]. As the frequency, duration, and intensity of heat waves are predicted to increase [12], research is increasingly focused on the impacts of heat waves on public health in urban areas.

It is well known that temperature has a great spatial variability in metropolitan areas because of the complex interaction of physical variables, such as surface and air temperature gradients, turbulent transport, and surface energy exchange [13]. Therefore, it is essential to find out the spatial distribution of urban heat and locate the population groups most vulnerable to heat, and then put forward some useful guidelines to prevent harm. Several scholars have assessed and mapped the heat vulnerability of urban populations around the world using heat vulnerability indicators (HVI). As is shown in Table 1, most previous studies combined many vulnerability factors (e.g., age, race, income, house condition, health condition, etc.) into an aggregate heat vulnerability indicator by means of spatial overlay analysis. However, there are no standard conclusions for the weights of vulnerability factors, because the current public health knowledge related to heat waves is limited [6]. As a consequence, a variety of weighting approaches were used in spatial overlay analysis. In addition, no widely accepted standard exists for the selection of the spatial unit in a heat vulnerability assessment model, so most previous studies selected census tract or administrative region as the spatial unit on account of data availability.

**Figure 1:**
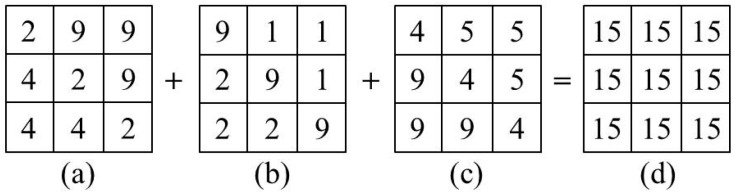
An illustration of the loss of information and spatial differentiation during aggregation. (**a**–**c**) are different vulnerability layers in a raster framework, which represent the spatial distribution of individual heat vulnerability factors such as age, race and income. The scores of all the heat vulnerability layers are reclassified to a range of 1 to 9. 1 represents the lowest risk level, while 9 means the highest risk level. (**d**) is the spatial aggregation result of all heat vulnerability factors.

Many factors lead to the inaccuracy of an aggregate heat vulnerability indicator. For example, equally fundamental dimensions and determinants of heat vulnerability are ignored because of lack of data [30,39]; there is no standard conclusion for the weights of multiple vulnerability layers when we aggregate them [27]; some useful information and spatial differentiation will be lost in the aggregation (Figure 1); the modifiable areal unit problem (MAUP) and statistical bias [27,40] will be produced when we aggregate other data to match the spatial unit of census data. In addition, different researchers have usually employed different heat vulnerability factors for reasons such as the difference of data availability and the subjective preferences of researchers [39], resulting in the lack of comparability among different studies.

In order to resolve these problems, this paper proposes a raster-based subdividing indicator to map urban heat vulnerability. We compared the results of this new indicator and other indicators, and discussed the following questions: (1) How can more detailed and accurate information about urban heat vulnerability be provided? (2) How can the comparability of urban heat vulnerability assessment results be enhanced among different studies?

## 2. Materials and Methods

### 2.1. Study Area

Sydney, located on Australia’s east coast, is the state capital of New South Wales (NSW) and the most populous city in Australia, with 5,005,400 residents in the Greater Sydney Statistical Division at the 2016 Census, up from 4,281,988 ten years earlier [41]. According to the observation data of Sydney (Observatory Hill) station, the Australian Bureau of Meteorology, and the definition of a heat wave by the World Meteorological Organization [25], Sydney has experienced five severe heat waves: in 1983, 1994, 2004, 2011 and 2015. Heat waves are becoming more frequent and are lasting longer in recent years [42].

The Australian Statistical Geography Standard (ASGS) provides a framework of statistical areas used by the Australian Bureau of Statistics (ABS) and other organizations to enable the publication of statistics that are comparable and spatially integrated. Statistical Areas Level 2 (SA2), one component of this statistical framework, was used to define the study areas in this research. 254 SA2s covering the greater metropolitan area of Sydney were selected as the study area in this research. The digital boundaries of all the 254 SA2s in Sydney are provided by ABS.

### 2.2. Study Design

The vulnerability framework developed by Turner et al. [43] and Wilhelmi et al. [44] was employed as the conceptual framework of heat vulnerability assessment in this study. In this conceptual framework, vulnerability was defined as a function of three interactive components: exposure, sensitivity and adaptive capacity [44], and each component consists of a set of indicators. Many studies utilized this vulnerability framework to assess and map heat vulnerability worldwide [26,29,31].

**Figure 2:**
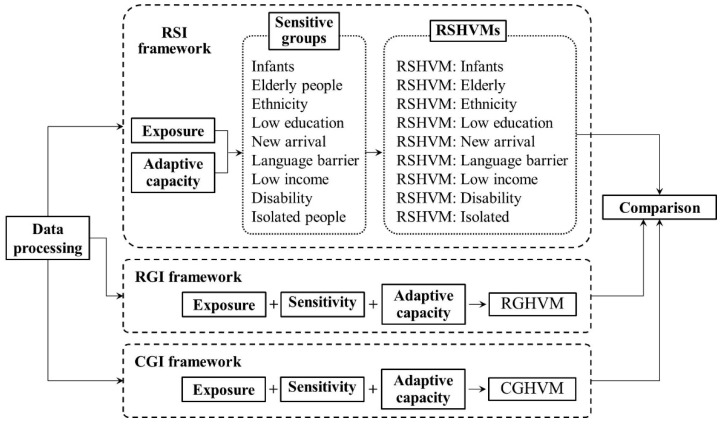
Flowchart of this research. (1) We assess and map urban heat vulnerability in 3 frameworks, and then compare and analyze the spatial differentiation among 3 heat vulnerability maps. (2) Abbreviations: RSI: raster-based subdividing indicator; RGI: raster-based general indicator; CGI: census unit-based general indicator; RSHVM: raster-based subdividing heat vulnerability map; RGHVM: raster-based general heat vulnerability map; CGHVM: census unit-based general heat vulnerability map.

The main steps of this study were (Figure 2): (1) to assess urban heat vulnerability by three components: exposure, sensitivity, and adaptive capacity (Table 2); (2) to assess and map urban heat vulnerability with three frameworks separately: raster-based subdividing indicator (RSI) framework, raster-based general indicator (RGI) framework, and census unit-based general indicator (CGI) framework. The following maps were obtained respectively: raster-based subdividing heat vulnerability maps (RSHVMs), raster-based general heat vulnerability map (RGHVM), and census unit-based general heat vulnerability map (CGHVM); (3) to examine the spatial relationships among RSHVMs, RGHVM and CGHVM by spatial statistic method; (4) to analyze the spatial accuracy of traditional CGHVM by the comparison of RSHVMs, RGHVM and CGHVM.

### 2.3. Indicators for Heat Vulnerability

According to previous studies and data availability, this research used the following heat vulnerability indicators (Table 2):

#### 2.3.1. Exposure

Air temperature data are commonly obtained from meteorological stations that operated by government. Those stations are usually unmovable and sparse, especially in the countryside. Therefore, air temperature data is inadequate for capturing the temperature gradient for a large area, and usually led to underestimation of the temperature effects [46]. Compared with air temperature data, remote sensing based land surface temperature (LST) data offer more spatial details of temperature. Although LST data cannot directly represent air temperature, many studies have shown the strong correlation between these two disparate data [47,48], so the applications of LST in heat vulnerability assessment are on the increase [15,16,27]. For example, Luis used a 3-day average LST data [31], and Ho used a single day LST data [38] as heat exposure indicator.

**Figure 3:**
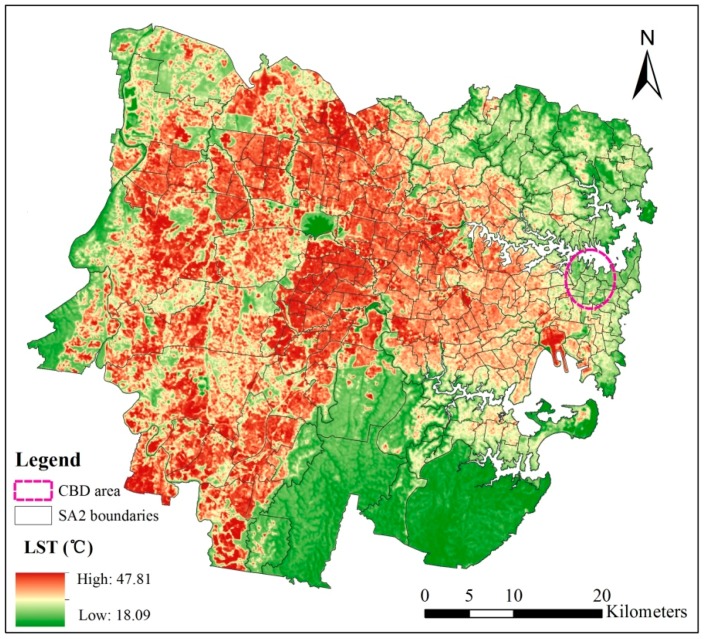
Land surface temperature (LST) of Sydney. CBD: Central Business District; SA2: Statistical Areas Level 2.

There are only 17 meteorological stations located in the study area (2855.77 km^2^), that is not enough for capturing the temperature gradient in Sydney. Therefore, in this study, a land surface temperature map with a spatial resolution of 30 m for Sydney has been derived as a 3-day average (29 December 2015; 30 January 2016; 16 January 2017) using Landsat 8 satellite images taken during hot summer days (Figure 3). Generalized single-channel method was used for land surface temperature retrieval from Landsat 8 imagines [49].

(1) $$\;T_{s}=\gamma \left[\frac{1}{\varepsilon }\left(\varphi_{1}L_{sensor}+\varphi_{2}\right)+\varphi_{3}\right]+\delta \;$$

where *T_s_* is land surface temperature; *ε* is the surface emissivity; *L_sensor_* is the at-sensor radiance from digital number (DN) data of TIRS-1 band. *γ* and *δ* are two parameters of the Planck function given by:

(2) $$\gamma \approx \frac{{T_{sensor}}^{2}}{b_{\gamma }L_{sensor}};\;\delta \approx T_{sensor}-\frac{{T_{sensor}}^{2}}{b_{\gamma }}$$

where *b_γ_* = *c*_2_/*λ* (1324 for TIRS-1); *T_sensor_* is the at-sensor brightness temperature which can be obtained from inversion of Planck’s law according to:

(3) $$\;T_{sensor}=\frac{c_{2}}{\lambda \ln\left(\frac{c_{1}}{\lambda^{5}L_{sensor}}+1\right)}\;$$

where *λ* is the radiation wavelength (10.904 μm for TIRS-1); *c*_1_ and *c*_2_ are the Planck radiation constants, with values of 1.19104 × 10^8^ W·μm^4^·m^−2^⋅SR^−1^ and 14,387.7 μm·K, respectively.

*φ*_1_, *φ*_2_, and *φ*_3_ in the Formula (1) are the parameters of the atmosphere functions, which can be calculated by the following Formula:

(4) $$\;\left[\begin{array}{c} \varphi_{1}\\ \varphi_{2}\\ \varphi_{3} \end{array}\right]=\left[\begin{array}{ccc} 0.04019 & 0.02916 & 1.01523\\ -0.38333 & -1.50294 & 0.20324\\ 0.00918 & 1.36072 & -0.27514 \end{array}\right]\left[\begin{array}{c} w^{2}\\ w\\ 1 \end{array}\right]\;$$

where *w* is the total atmospheric water vapor content, which can be derived from Moderate Resolution Imaging Spectroradiometer (MODIS) Near-Infrared Total Precipitable Water Product (MODIS 05) provided by National Aeronautics and Space Administration (NASA).

#### 2.3.2. Sensitivity

Specific population groups are more vulnerable to heat waves for specific reasons. For example, populations above the age of 65 have a higher mortality risk and hospital admission rate for respiratory and other heat-related diseases on hot days [2,50]; low income people and people with low levels of education have been shown to be highly associated with increased heat stress, mortality, and increased risk of heat-related morbidity [51,52]; many studies also have shown that some populations like people with language barriers, people living alone and new immigrants are more vulnerable to heat [9,15,28]. In this research, 9 of these fragile groups have been selected as sensitivity indicators according to previous studies and data availability. 2016 census data provided by the Australian Bureau of Statistics (ABS) was used in this research.

**Figure 4:**
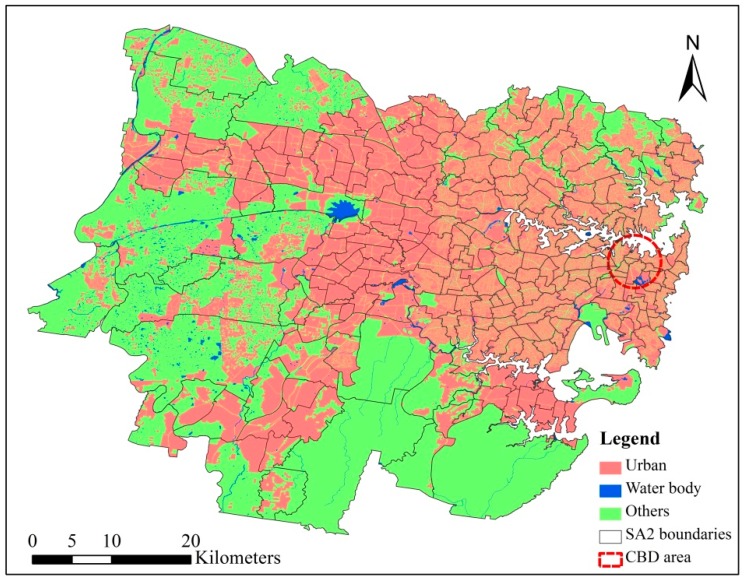
Land use types of the study area. “Water body” includes the land use classes of “river and drainage system” and “wetland” in Land Use Mapping Program (LUMAP); “Urban region” includes the land use classes of “urban” in LUMAP. CBD: Central Business District; SA2: Statistical Areas Level 2.

To account for spatial concentration patterns, 11 census unit-based indicators were expressed in density terms (per unit of each pixel) using the urban region of each census tract. This normalization method prevents the generation of spatial biases induced by the size of the census unit [31]. New South Wales (NSW) Landuse 2013 dataset [53] was used to extract the urban region of Sydney (Figure 4). NSW Landuse 2013 dataset was published by the NSW government on 20 December 2017, and its metadata template type is vector. It provides the land use map of NSW according to the classification scheme of NSW Land Use Mapping Program (LUMAP). The land use classes of LUMAP include conservation area, cropping, grazing, horticulture, intensive animal production, mining and quarrying, power generation, river and drainage system, special category, transport and other corridors, tree and shrub cover, urban, and wetland.

#### 2.3.3. Adaptive Capacity

Four indicators have been selected to determine adaptive capacity to heat vulnerability according to previous studies and data availability. (1) Traffic convenience. Traffic conditions are important for people in high vulnerability groups seeking cooling on a hot summer day [2]. In this research, the density of dwellings having more than one motor vehicle was used to represent traffic convenience. People with motor vehicles have higher capacity to relieve heat stress, because they can go to hospital, green space or a water body much easier and quicker. The data source of this indicator is the 2016 census data provided by ABS; (2) Internet access. People with home internet access have a higher capacity to obtain more heat-related information or guides from local government or other members of the public [31]. The data source of this indicator was also provided by the ABS; (3) Proximity to woody vegetation. Trees can effectively mitigate outdoor thermal stress in urban environments [10]. The NSW Woody Vegetation Extent 2011 dataset was used in this study. This dataset was published by the NSW government [54] on 2 April 2015. It is a state-wide binary classification of woody vegetation derived from multi-temporal SPOT-5 satellite imagery at a 5 m spatial resolution. This latest open access map of the extent of woody vegetation for NSW is the highest detail to date. It shows the location and extent of woody vegetation in NSW for the year 2011. This woody dataset was resampled at 30 m spatial resolution by the same cubic resampling technique, which is consistent with LST layer (30 m); Focal statistics was used to calculate for each pixel location a statistic of woody pixels within a 3 km × 3 km circle neighborhood around it. A bigger output value of a pixel means more woody pixels around it, and has higher capacity to relieve heat stress. (4) Proximity to water bodies. Previous studies found that urban wetlands create a cool-island effect in warm seasons [55]. Water bodies of Sydney have been extracted from Landsat 8 imagine by Modification of the Normalized Difference Water Index (MNDWI) [56]. Similar resample and focal statistics procedures were applied for the water body dataset as the woody dataset.

## 3. Calculations

Calculations of three heat vulnerability indicators (HVI) are as follows, and Arcgis 10.0 software (Esri, Redlands, CA, USA) was used for all the calculations.

### 3.1. Raster-Based Subdividing Indicator (RSI)

HVI of each fragile group at pixel scale should be calculated in a raster-based subdividing indicator (RSI) framework. All indicators were normalized by the following Formula:

(5) $$\;Y_{i}=100\times \frac{x_{i}-x_{i}min}{x_{i}max-x_{i}min}\;$$

where *Y_i_* is the normalized value of *x_i_* ranged within (0,100); *x_i_* is the original value of pixel *i*; *x_i_max* is the maximum value of *x_i_*; while *x_i_min* is the minimum value of *x_i_*.

The heat vulnerability indicators (HVIs) of different fragile groups at pixel scale were calculated by the following Formula [31]:

(6) $${\mathrm{HVI}}_{j}=E+S_{j}-\frac{\sum_{i=1}^{4}A_{i}}{4};\;j=1,\ldots ,9;\;i=1,\ldots ,4$$

where HVI*_j_* is the HVI of fragile group *j*; *E* is the exposure level (land surface temperature); *S_j_* is the sensitivity level of fragile group *j*; and *A_j_* corresponds to the level of adaptive capacity layer *i*.

Finally, the results of HVIs were normalized by Formula (5) again to obtain the heat vulnerability indicators ranging from 0 to 100 [57]. 

### 3.2. Raster-Based General Indicator (RGI)

The general heat vulnerability indicator (GHVI) of each pixel was calculated in a raster-based general indicator (RGI) framework. The GHVI of each pixel was calculated by the following formula:

(7) $$\mathrm{GHVI}=E+\frac{\sum_{j=1}^{9}S_{j}}{9}-\frac{\sum_{i=1}^{4}A_{i}}{4};\;j=1,\ldots ,9;\;i=1,\ldots ,4$$

where *E* is the exposure level (land surface temperature); *S_j_* is the sensitivity level of the fragile group *j*; and *A_j_* corresponds to the level of adaptive capacity layer *i*.

### 3.3. Census Unit-Based General Indicator (CGI)

The general heat vulnerability indicator (GHVI) of each SA2 was calculated in the census unit-based general indicator (CGI) framework. 

Zonal statistics was used to calculate the average value of 3 raster-based indicators within the zones of respective SA2s: land surface temperature, proximity to woody vegetation, and proximity to water bodies. The other 11 census unit-based indicators of each SA2s were calculated directly. Formula (7) was used to calculate the GHVI of each SA2. The final results of the GHVI were normalized by Formula (5) to obtain the heat vulnerability indicator ranging from 0 to 100. 

## 4. Results

### 4.1. Raster-Based Subdividing Heat Vulnerability Map (RSHVM)

**Figure 5:**
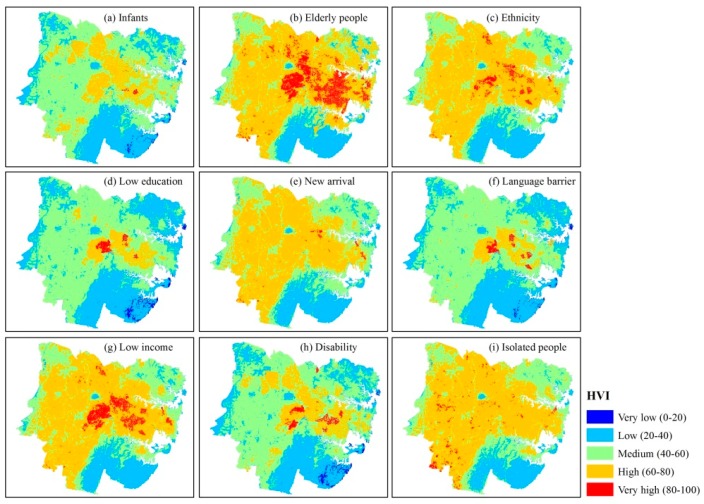
Raster-Based Subdividing Heat Vulnerability Map (RSHVM) of each fragile group in Sydney. Equal interval classification method was used to ensure the comparability among heat vulnerability indicators (HVIs).

Figure 5 presents the RSHVM of each fragile group in Sydney.

**Figure 6:**
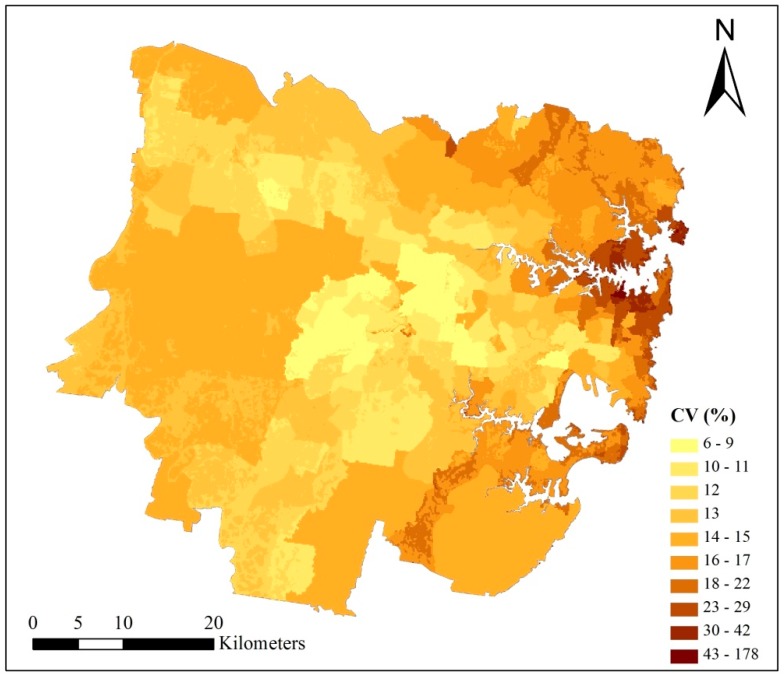
Coefficient of variation (CV) of 9 HVI scores at pixel scale. A total of 9 HVI scores of each pixel were presented in Figure 5. The formula of CV is: CV_i_ = (STD_i_/MEAN_i_) × 100%, where CV_i_ is the coefficient of variation of pixel i; STD_i_ is the standard deviation of 9 HVIs of pixel i; MEAN_i_ is the average value of 9 HVIs of pixel i.

As is shown in Figure 5, there are some differences among the heat vulnerability maps (HVMs). (1) The HVI scores of 9 fragile groups are different. The average HVI score of “Elderly people” is up to 61.65, which is the highest among 9 fragile groups; while people categorized as “Low education” had the lowest average HVI score of 44.41. This is possibly because there is a large elderly population in this region, which accounted for 13.32% of the total population; while the low education category accounted for just 4.55% of the total population. (2) There is conspicuous spatial variability among 9 fragile groups. The coefficients of variation (CV) of the 9 HVI scores for each pixel have been calculated and mapped in Figure 6. The average value of CV is 13.43% and the maximum is up to 178.24%, which indicates that there are significant differences among those 9 HVI scores at the pixel scale. As is shown in Figure 6, higher CV pixels are mainly located in the central business district (CBD) of Sydney and its surrounding areas, such as Woolloomooloo, Pyrmont and Ultimo. These are areas where there is public housing adjacent to areas of wealth, therefore the demographic structure of these areas are quite different and complicated, resulting in large differences among 9 HVI scores.

### 4.2. Raster-Based General Heat Vulnerability Map (RGHVM)

**Figure 7:**
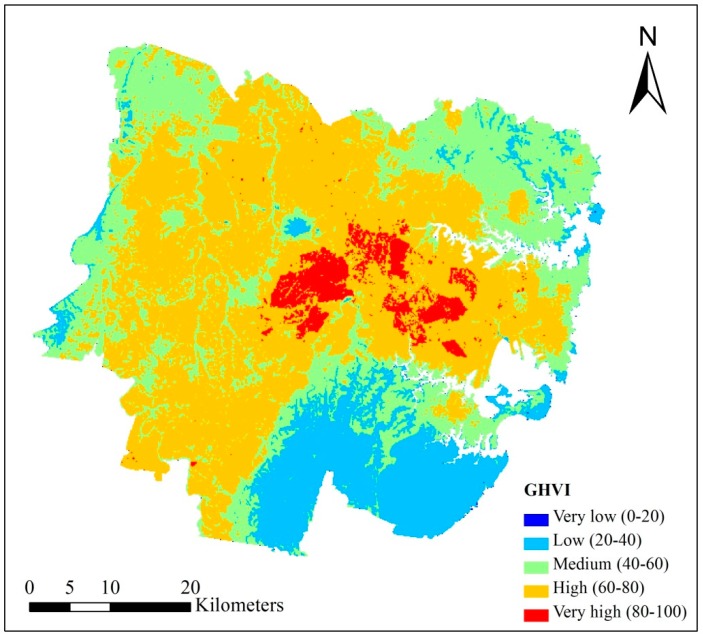
Raster-Based General Heat Vulnerability Map (RGHVM) in Sydney.

Figure 7 presents the Raster-Based General Heat Vulnerability Map (RGHVM) in Sydney. As is shown in Figure 7, (1) very high HVI pixels are mainly located in the middle part of the study area, such as Liverpool, Strathfield, Parramatta and Auburn. These areas have a large population, often in higher density dwellings, and have a relatively high temperature being away from the ocean. (2) The CBD of Sydney is located near Sydney Harbour, which has less very high HVI pixels than the middle part of the study area. The possible reasons are the CBD area has better infrastructure, higher income, and lower temperature due to proximity to the ocean. (3) The southern part of the study area has the lowest HVI scores per pixel. The reason is Royal National Park and Heathcote National Park are located in this area. This area is therefore rich in vegetation, low in population, close to the ocean and has the lowest heat vulnerability.

### 4.3. Census Unit-Based General Heat Vulnerability Map (CGHVM)

**Figure 8:**
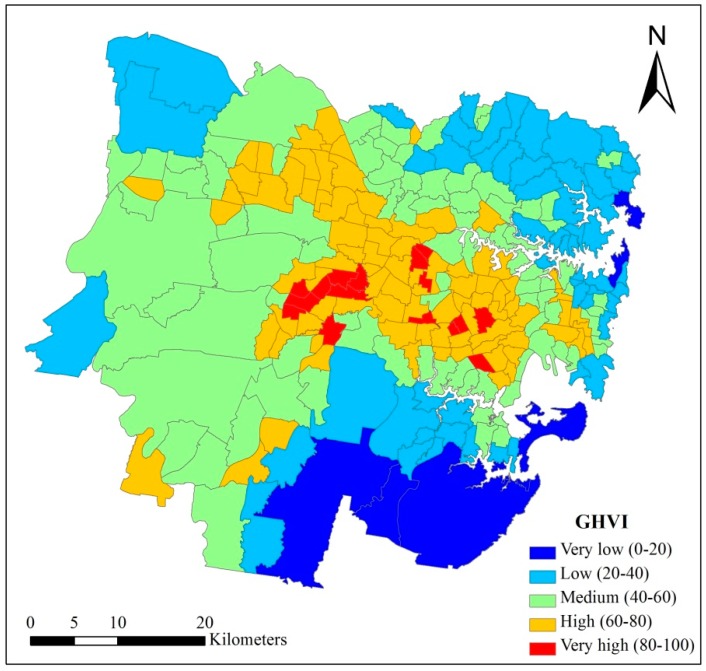
Census unit-based General Heat Vulnerability Map (CGHVM) in Sydney.

Figure 8 presents the Census unit-based General Heat Vulnerability Map (CGHVM) in Sydney. As is shown in Figure 8, the spatial pattern of CGHVM is similar to the RGHVM. In CGHVM, high HVI areas are mainly located in the middle and western part of the study area, while the HVI scores of the northeastern and southern areas are relatively low. However, obviously, CGHVM cannot reflect the spatial diversity of heat vulnerability within each SA2 as RGHVM.

Table 3 presents the statistical results of all pixels’ GHVI scores in some SA2s. As is shown in Table 3, the GHVI scores of all the pixels in one SA2 vary greatly, and the maximum difference is up to 67.92 (81.23 − 13.31 = 67.92) which appeared in Rosemeadow-Glen Alpine. Table 3 also indicated that statistical bias existed in many SA2s when we assume that 80 is the threshold for very high vulnerability areas [35]. For example, the minimum GHVI score in Canley Vale-Canley Heights is 58.09, which is much lower than 80, but it will be treated as very high heat vulnerability area in the CGI framework because the average GHVI score of Canley Vale-Canley Heights is higher than 80. Conversely, although the GHVI scores of many pixels in Lidcombe are higher than 80, it will be ignored in the CGI framework because the average GHVI score of Lidcombe is lower than 80. 

### 4.4. Comparison of RGHVM and RSHVM

The comparison results of the raster-based general heat vulnerability map (RGHVM) and the raster-based subdividing heat vulnerability maps (RSHVMs) are as follows: 

The GHVI score of each pixel was subtracted by the nine HVI scores separately and the results were summarized in Table 4. As is shown in Table 4, there are obvious variations among the GHVI scores and the nine HVI scores at the pixel scale. The difference of “Low education” is the biggest in the nine fragile groups, with an average variation of 13.31.

Table 5 presents the HVI score and the GHVI score of some typical SA2s. As is shown in Table 5, the HVI scores of different fragile groups vary, and the maximum difference of up to 54.20 (82.01 − 27.81 = 54.20) appeared in the harborside area of Potts Point–Woolloomooloo (where the former has high-income residents, whereas the latter area is home to many public housing tenants). Table 5 also indicated that the GHVI scores of all the listed SA2s are below 80 even when the HVI scores of some fragile groups are much bigger than 80. In other words, those SA2s will be ignored by decision makers under the CGI framework. 

**Figure 9:**
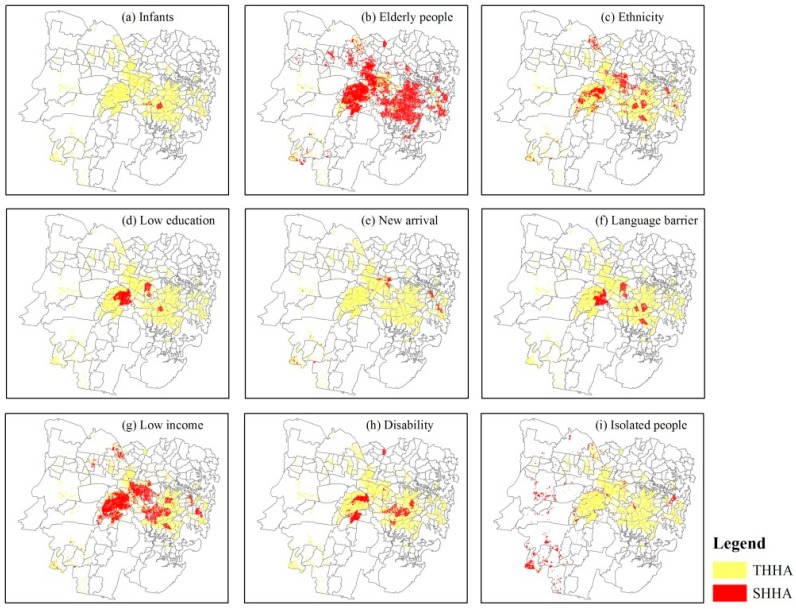
Subdividing the high HVI score area (SHHA) of each fragile group in Sydney. The THHA layer is lower than the SHHA layer, so the THHA is invisible if there is SHHA.

**Figure 10:**
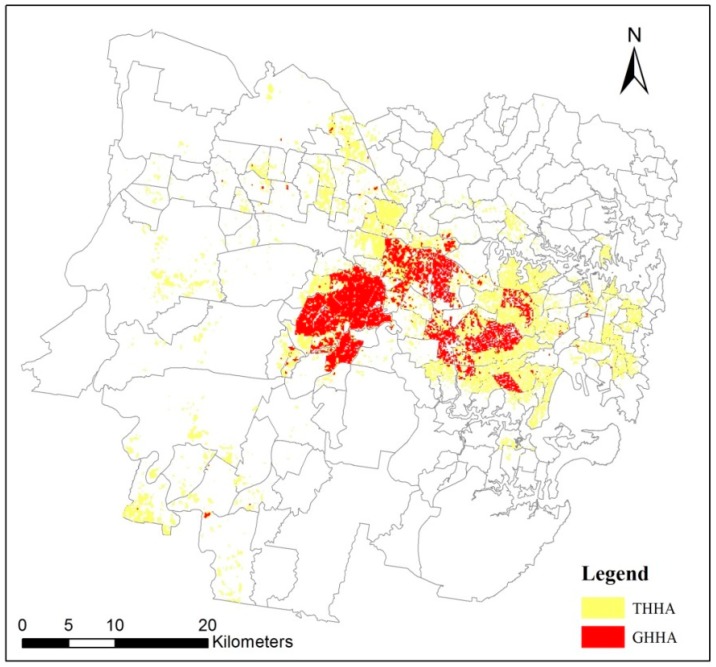
General high HVI score area (GHHA) of Sydney.

The very high HVI area has been of primary concern in previous research [2,58], so we explored the spatial stability of RSHVMs and RGHVM in very high HVI areas. A very high HVI pixel was defined as a pixel if it has one or more HVI scores or GHVI score bigger than 80 [35]. 386,400 pixels were selected according to this definition, and the total area of those 386,400 pixels is named as the total very high HVI area (THHA). Correspondingly, the very high HVI area of each fragile group is named as the subdividing very high HVI area (SHHA), and the very high GHVI area is named as the General high HVI area (GHHA). Obviously, both of SHHA and GHHA are the subsets of THHA. SHHA of each fragile group were extracted and mapped in Figure 9, and GHHA was extracted and shown in Figure 10.

As is shown in Figure 9 and Figure 10, the size and spatial distribution of SHHAs and GHHA are quite different. Pixel numbers of total very high HVI area (THHA), subdividing very high HVI areas (SHHAs) and general very high HVI area (GHHA) are counted and presented in Table 6. As is shown in Table 6, GHHA just extracted 113,088 very high HVI pixels out of the total 348,384 pixels, so 235,296 (348,384 − 113,088 = 235,296) very high HVI pixels were ignored in GHHA, which account for 67.54% of the THHA.

### 4.5. Comparison of RSHVM and CGHVM

**Figure 11:**
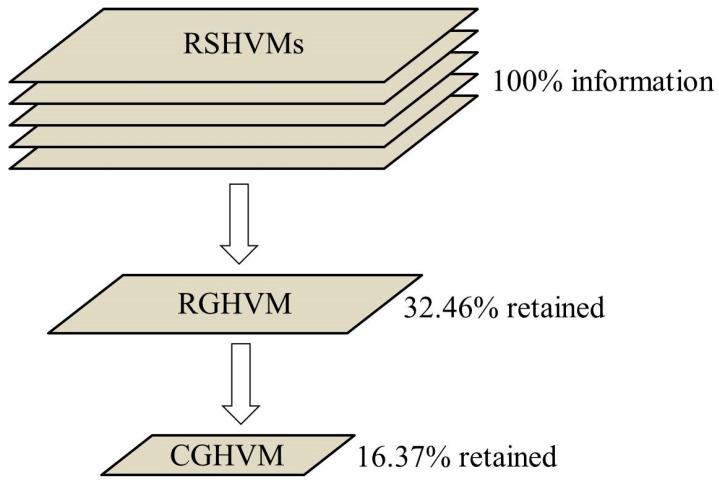
Information retained in three kinds of heat vulnerability maps.

We compared the very high HVI areas of the raster-based subdividing heat vulnerability maps (RSHVMs) and the census unit-based general heat vulnerability map (CGHVM) too. A very high HVI pixel in RSHVM was defined as a pixel if it has one or more HVI scores bigger than 80; while a very high GHVI SA2 in CGHVM was defined as a SA2 region whose GHVI score is bigger than 80 [35]. 16 SA2s were extracted according to this definition. There are 68,368 pixels located in those 16 very high GHVI SA2s. The statistical results of those 68,368 pixels show that there are 57,024 very high HVI pixels which have one or more HVI scores or a GHVI score bigger than 80. Therefore, the correct extract ratio of very high HVI pixels in CGHVM is just 16.37% (57,024/348,384 × 100%). There are 291,360 very high HVI pixels remaining outside of those 16 very high GHVI SA2s, which means up to 83.63% of useful heat vulnerability information was ignored in CGHVM (Figure 11).

## 5. Discussion

This study provides a new raster-based subdividing indicator (RSI) framework to assess and map urban heat vulnerability. Compared with traditional census unit-based general indicator (CGI) [59], the RSI framework could provide more useful and accurate spatial information of heat vulnerability to decision makers. That information enables local governments to identify hot spots of heat vulnerability and allocate resources and assistance to target areas where the most fragile groups are gathered [29]. 

### 5.1. The Merits of RSI Frameworkthe Results of RSI Framework Are More Accurate than Traditional CGI Framework. The Reasons Are as Follows

(1) The spatial variation information of each fragile group was provided in the RSI framework. In fact, different fragile groups usually need different resources and assistance during heat waves [60]. For example, it’s suitable to provide additional emergency ambulance facilities and health care workers for infants and elderly people who have poor physical resistance to heat; while initially multi-language heat information and guidelines are more useful to overcome language barriers. As for low income people, perhaps the most effective way to aid adaption to heat waves is through ease of access to public ‘cool spots’ like well shaded parks, water bodies and libraries, although research by Sampson et al. [61] explains the various socio-technical complexities to such an approach. However, the CGI framework cannot provide that information.
(2) Aggregate error will be avoided in the RSI framework. A sensitivity indicator is usually aggregated by many factors (such as infants, the elderly, and people of low income) under the CGI framework [31]. However, this aggregation not only leads to the inaccuracy of useful spatial information, but also may mislead decision makers (Figure 1). As is shown in Table 5, it is possible that some census units with high vulnerability groups will be ignored by decision makers under the CGI framework. However, because there is no need to aggregate the multiple sensitivity factors into a combined indicator under the RSI framework, the aggregate error will be avoided.
(3) Weighting problems will be avoided in the RSI framework. Many scholars admitted that they use equal weights or principal component analysis (PCA) to combine multiple sensitivity factors, because no information exists from which a more appropriate weighting can be derived [27,31,57]. This is a possible source of indicator inaccuracy [59]. However, there is no need to calculate a combined sensitivity indicator under the RSI framework, so the weighting problems will be avoided.
(4) Modifiable areal unit problem (MAUP) problem and statistical bias will be avoided in the RSI framework. Census information is an important data source for mapping urban heat vulnerability, and it is usually counted with some specific spatial unit like postal code and census tract. In order to match spatial units employed by the census, most heat vulnerability studies use a spatial statistic method such as zoning or scaling to process the base data. This data processing procedure not only leads to coarse spatial resolution of heat vulnerability assessment results, but also causes the modifiable areal unit problem (MAUP) and statistical bias (Table 3). However, those problems will be avoided under the RSI framework.

The RSI framework has higher flexibility and extensibility than the traditional CGI framework. Usually, all the factors will be aggregated to a combined heat vulnerability indicator under the CGI framework [31]. Therefore, if the data of new fragile groups becomes available, we need to renew the whole model and map the heat vulnerability again. However, the assessing and mapping results of different fragile groups are independent to each other under the RSI framework. In this case, it is possible to assess and map the heat vulnerability of new fragile groups when data becomes available, and it’s unnecessary to modify the previous mapping results. For this reason, the RSI framework is more flexible and much easier to extend than the CGI framework.

The RSI framework also has higher comparability than traditional CGI framework. Lacking comparable data and results is still a challenge in mapping heat vulnerability research [62]. In previous urban heat vulnerability research, Land surface temperature (LST) data derived from satellite images or meteorological data released by local government are usually used as an exposure indicator. Green space, water bodies and road networks derived from satellite images are usually used as the data sources of an adaptive capacity indicator. Therefore, both the exposure indicator and the adaptive capacity indicator can be easily accessed and are comparable if the data processing standards have been unified. However, sensitivity data is usually based on census data released by local governments, and the statistical standards and contents are non-uniform among different countries and regions. Due to data availability, research papers can substantially vary in the number of fragile groups assessed; for instance, Dong et al. [25] includes only two fragile groups, while Johnson et al. [20] includes 14 fragile groups. Because all the sensitivity factors need to be aggregated to a combined heat vulnerability indicator under the CGI framework, the non-uniform sensitivity data leads to the incomparable mapping results of heat vulnerability.

Although the condition of non-uniform sensitivity data still exists under the RSI framework, the comparability will be greatly enhanced if we just compare the heat vulnerability map (HVM) of one specific fragile group. For example, if the HVMs of two fragile groups (the elderly and infants) are obtained in region A, while the HVMs of ten fragile groups (the elderly, infants and others) are mapped in region B, the HVMs of elderly people and infants are comparable between region A and B under the RSI framework. Furthermore, if more data in region A becomes available in the future, the HVMs of more fragile groups will become comparable between region A and B. Therefore, the comparability of studies in different locations will be enhanced under the RSI framework.

### 5.2. Limitations of This Study

Despite its strengths, this study has several limitations. (1) The results of this study have not been validated by local health data (such as emergency hospital admissions, morbidity and mortality data) since it is not readily available. It is widely known that validation is an important component in mapping urban heat vulnerability [63], but many heat vulnerability studies did not validate their results due to a lack of data [26,30,35]. However, some studies have validated the heat vulnerability indicator (HVI) by using local health data, and their results show that the HVI offered potential as an *a priori* indicator of the level of ambulance callout and mortality for all summer days and heat wave events [63,64]. (2) There may also be some controversy in the selection of sensitive indicators. For example, population density was commonly used in previous studies [8,25]. However, it is not suitable to select population density under the RSI framework because 9 sensitivity indicators have already been expressed in density terms independently. In addition, some other sensitive indicators are not selected in this study due to data availability, such as old dwellings without air conditioning, or the existence of homeless people. Nevertheless, most of the commonly used sensitive indicators were selected in this study. (3) This study utilized multiple data sources with different collection dates. Such as 2016 census data, 2015–2017 land surface temperature (LST) data, New South Wales (NSW) Landuse 2013 dataset, NSW Woody Vegetation Extent 2011 dataset. We admit that the discrepancy among different data sources inevitably created some temporal ambiguity during the modeling process. We have noticed that MODIS satellite imagery could provide daily LST, land cover type, and NDVI products during the research period. However, the spatial resolutions of those products (250 m) are much coarser than the datasets provided by NSW government (5 m), so we selected the present datasets in this study.

## 6. Conclusions

In the context of global climate change and urban heat island effects, the urban heat vulnerability indicator and associated vulnerability mapping has developed significantly during the past decade. However, the heat vulnerability assessment models are still in the early stages of development, and many aspects, including indicator selection, data availability, indicator weighting and aggregation, are all possible sources of indicator inaccuracy.

This research created a raster-based subdividing indicator (RSI) to map urban heat vulnerability, and compared it with a raster-based general indicator (RGI) and a census unit-based general indicator (CGI). Results show that: (1) compared with the RSI framework, 67.54% of very high heat vulnerability pixels were ignored in the RGI framework; and up to 83.63% of very high heat vulnerability pixels were ignored in the CGI framework. (2) Compared with the previous CGI framework, the RSI framework has many advantages, such as more accurate results, more flexible model structure, and higher comparability among different studies.

With the rapid development of remote sensing (RS) and geographic information system (GIS) technology, the availability of high resolution spatial data is increasing, and the data processing ability of computer systems is growing. In combination, this has created the possibility for mapping urban heat vulnerability in a RSI framework. Considering the merits of the RSI framework, we have reason to believe that it will lay a solid foundation for future urban heat vulnerability studies and provide more useful and accurate information to decision makers.

## Figures and Tables

**Table 1:** Basic information of previous heat vulnerability studies.

Author and Year	Study Area	Spatial Unit	Number of Fragile Groups	Approach
Vescovi et al., 2005 [14]	Quebec, Canada	census subdivision	4	Overlay/EW
Reid et al., 2009 [1]	USA	census tract	7	Overlay/PCA
Rinner et al., 2010 [15]	Toronto, Canada	census tract	12	Overlay/MCA
Tomlinson et al., 2011 [16]	Birmingham, UK	641 census areas	2	Overlay/EW
Hondula et al., 2012 [17]	Philadelphia, USA	zip code area	8	Overlay/PCA
Chuang, 2012 [18]	Phoenix, USA	census tract	4	Overlay/EW
Loughnan et al., 2012 [19]	Melbourne, Australia	postal area	3	Overlay/RA
Johnson et al., 2012 [20]	Chicago, USA	census block group	14	Overlay/PCA
Wolf et al., 2013 [8]	London, UK	4765 census districts	5	Overlay/PCA
Aubrecht et al., 2013 [21]	National Capital Region, USA	92,000 census blocks	5	Overlay/EW
Harlan et al., 2013 [22]	Maricopa, USA	census block group	7	Overlay/PCA
Depietri et al., 2013 [23]	Cologne area, Germany	85 districts	3	Overlay/EW
Maier et al., 2014 [24]	Georgia, USA	county	7	Overlay/PCA
Dong et al., 2014 [25]	Beijing, China	319 sub-districts	2	Overlay/EW
Zhu et al., 2014 [26]	Guangdong, China	124 counties	6	Overlay/ES
Loughnan et al., 2014 [7]	Australian capital cities	SA, LGA	4	Overlay/RA
Chak et al., 2015 [27]	Vancouver, Canada	60 m pixel	8	Overlay/EW
El-Zein et al., 2015 [28]	Sydney, Australia	15 LGA	6	Overlay/MCA
Weber et al., 2015 [29]	Philadelphia, USA	census block	4	Overlay/EW
Aminipouri et al., 2016 [2]	Vancouver, Canada	105 census areas	8	Overlay/RA
Macnee et al., 2016 [9]	Osaka, Japan	1904 census districts	6	Overlay/PCA
Li et al., 2016 [30]	Tibet, China	73 counties	5	Overlay/PCA
Luis et al., 2016 [31]	Santiago de, Chile	277 census tracts	6	Overlay/PCA
Christenson et al., 2017 [32]	Milwaukee and Wisconsin, USA	census block groups	7	Overlay/EW
Azhar et al., 2017 [33]	India	640 districts	7	Overlay/PCA
Kim et al., 2017 [34]	Korea	232 counties	5	Overlay/RA
Méndez-Lázaro et al., 2018 [35]	San Juan, Puerto Rico	227 census tracts	8	Overlay/EW
Voelkel et al., 2018 [36]	Portland, USA	census block group	6	Overlay/CA
Mushore et al., 2018 [11]	Harare, Zimbabwe	30 m pixel	4	Overlay/EW
Nayak et al., 2018 [37]	New York State, USA	2751 census tracts	9	Overlay/PCA
Ho et al., 2018 [38]	Canada	census district	8	Overlay/MCA

Notes: EW: equal weight; ES: expert survey; PCA: principal component analysis; MCA: Multi-criteria analysis; RA: regression analysis; CA: covariance analysis; SA: statistical local area; LGA: local government area.

**Table 2:** Heat vulnerability indicators of this research.

Item	Indicators	Detail	References Used Similar Indicator
Exposure	Land surface temperature (LST)	a 3-day average LST map of Sydney	[15,16,27]
Sensitivity	Infants	Density of infants (0–4)	[7,31]
	Elderly people	Density of elderly people (>65)	[3,30]
	Ethnicity	Density of people not born in Australian	[2,22]
	Low education people	Density of people with low levels of education	[9,15]
	New arrival	Density of people who arrived in Australian after 2014	[18,22]
	Language barrier	Density of people with dysfluent English	[7,28]
	Low income people	Density of people with low income	[11,27]
	Persons with disability	Density of people that core activity need for assistance	[31,45]
	Isolated people	Density of people usually living alone	[1,9]
Adaptive capacity	Traffic convenience	Density of dwelling have more than one motor vehicles	[7,31]
	Internet access	Density of dwelling have internet access from dwelling	[28,31]
	Proximity to woody vegetation	Number of woody pixels around each pixel	[2,9]
	Proximity to water bodies	Number of water body pixels around each pixel	[7,9]

**Table 3:** Basic statistics of some SA2s in CGHVM.

SA2	Pixel Counts	Basic Statistics of Pixels’ GHVI Scores in Each SA2					
		Minimum	Maximum	Mean	Standard Deviation	CV	Range
Manly-Fairlight	7328	6.97	52.17	35.87	10.29	28.70	45.20
Cronulla-Kurnell-Bundeena	27,584	4.79	56.38	36.60	9.35	25.54	51.60
Peakhurst-Lugarno	8208	11.71	72.96	58.78	9.26	15.75	61.25
Chipping Norton-Moorebank	16,000	37.08	81.05	63.47	10.19	16.05	43.96
Rosemeadow-Glen Alpine	53,440	13.31	81.23	63.88	8.25	12.92	67.92
Cabramatta-Lansvale	8512	37.69	92.18	78.06	12.43	15.93	54.50
Canley Vale-Canley Heights	6016	58.09	89.68	83.03	6.52	7.85	31.59
Auburn-South	2688	54.43	90.71	79.15	6.38	8.07	36.28
Lidcombe	7120	53.49	90.11	75.43	5.04	6.68	36.63
Liverpool	7040	54.26	90.57	82.12	5.84	7.11	36.31

**Table 4:** Basic statistics for the difference between the general heat vulnerability indicator (GHVI) and the HVIs at pixel scale.

Item	Minimum	Maximum	Mean |D|	Standard Deviation
GHVI-HVI (Infants)	−13.78	25.70	10.04	3.67
GHVI-HVI (Elderly)	−36.26	14.89	4.08	4.31
GHVI-HVI (Ethnicity)	−17.89	9.71	1.97	2.13
GHVI-HVI (Low education)	−4.27	34.23	13.31	3.72
GHVI-HVI (New arrival)	−17.89	29.80	3.56	4.81
GHVI-HVI (Language barrier)	−12.88	31.60	12.24	4.02
GHVI-HVI (Low income)	−20.56	14.21	1.82	1.38
GHVI-HVI (Disability)	−17.73	30.15	10.74	4.27
GHVI-HVI (Isolated)	−39.35	26.25	4.29	4.38

Note: |D| is the absolute value of difference between GHVI and HVIs of each pixel; while Mean |D| is the average value of all pixels’ |D|.

**Table 5:** HVI scores and GHVI score of some SA2s.

SA2	Average HVI Score of Each SA2									General HVI
	Infants	Elderly	Ethnicity	Low Education	New Arrival	Language Barrier	Low Income	Disability	Isolated	
Potts Point-Woolloomooloo	33.79	77.70	52.19	28.97	42.49	27.81	43.07	57.10	**82.01**	52.50
Castle Hill-East	48.70	87.68	63.38	46.23	58.16	46.91	64.61	78.59	65.17	67.44
Woollahra	49.78	83.32	51.76	34.15	45.18	33.76	51.03	46.44	56.29	53.84
Ashfield	61.46	85.64	76.24	60.76	67.55	63.32	76.11	74.97	70.63	77.05
Kensington (NSW)	54.36	74.65	73.07	46.29	74.41	49.00	85.11	54.69	64.41	68.55
Croydon Park-Enfield	60.29	85.02	71.71	61.89	64.03	58.63	74.83	67.96	70.92	74.34
Belmore-Belfield	62.48	83.99	74.15	67.33	66.61	63.96	77.59	72.75	72.61	50.88
Concord-Mortlake-Cabarita	57.06	83.27	65.76	54.46	58.93	51.25	68.59	61.05	65.30	68.16
Pyrmont-Ultimo	55.04	67.58	82.87	42.54	82.87	63.75	82.42	45.49	63.69	69.65
Bondi Junction-Waverly	55.85	82.33	59.95	39.20	52.20	39.40	57.11	61.59	60.02	60.97

**Table 6:** Pixel counts of THHA, SHHAs and GHHA.

Item	Pixel Counts	Percent
SHHA: Infants	4544	1.30%
SHHA: Elderly	276,192	79.28%
SHHA: Ethnicity	70,768	20.31%
SHHA: Low education	30,512	8.76%
SHHA: New arrival	12,080	3.47%
SHHA: Language barrier	31,088	8.92%
SHHA: Low income	144,848	41.58%
SHHA: Disability	44,896	12.89%
SHHA: Isolated	46,768	13.42%
GHHA	113,088	32.46%
THHA	348,384	100.00%

Notes: “Percent” is defined as SHHA/THHA × 100% and GHHA/THHA × 100% respectively.

## Abbreviations

ABS	Australian Bureau of Statistics
CGI	census unit-based general indicator
GHVI	general heat vulnerability indicator
HVM	heat vulnerability map
RGHVM	raster-based general heat vulnerability map
RSHVM	raster-based subdividing heat vulnerability map
SA2	statistical areas level 2
THHA	total very high HVI area
CGHVM	census unit-based general heat vulnerability map
GHHA	general very high HVI area
HVI	heat vulnerability indicator
MAUP	modifiable areal unit problem
RGI	raster-based general indicator
RSI	raster-based subdividing indicator
SHHA	subdividing very high HVI area
